# Clinical observation and risk assessment after splenectomy in hepatolenticular degeneration patients associated with hypersplenism

**DOI:** 10.3389/fsurg.2022.972561

**Published:** 2022-09-23

**Authors:** Wanzong Zhang, Qingsheng Yu, Hui Peng, Zhou Zheng, Fuhai Zhou

**Affiliations:** ^1^ First Affiliated Hospital of Anhui University of Traditional Chinese Medicine, Hefei, China; ^2^ Anhui Academy of Chinese Medicine, Hefei, China

**Keywords:** hepatolenticular degeneration, hypersplenism, splenectomy, liver function, complication

## Abstract

**Background:**

Both hepatolenticular degeneration (HLD) and viral hepatitis B (HBV) can cause hypersplenism, but whether splenectomy is needed or can be performed in HLD patients associated with hypersplenism is still controversial. At present, HLD combined with hypersplenism has not been listed as the indication of splenectomy.

**Objective:**

This study aimed to investigate the efficacy, risks, and postoperative complications of splenectomy in HLD patients associated with hypersplenism.

**Methods:**

We retrospectively analyzed the clinical data of 180 HLD patients with hypersplenism who underwent splenectomy in the Department of General Surgery, First Affiliated Hospital of Anhui University of Traditional Chinese Medicine, from January 2001 to December 2015. To evaluate the efficacy of splenectomy, the hemogram of white blood cells (WBC), red blood cells (RBC), platelets (PLT), and the liver function indexes including alanine aminotransferase, aspartate aminotransferase, and total bilirubin were recorded before surgery and 1, 3, 5, 7, and 14 days after surgery. In addition, the clinical data of 142 HBV patients with hypersplenism who underwent splenectomy over the same period were also recorded and compared with that of HLD patients. In particular, aiming to assess the risks of splenectomy in HLD, we also compared postoperative complications and 36-month mortality between the two groups.

**Result:**

The level of WBC, RBC, and PLT were all elevated after splenectomy in both the HLD group and the HBV group. However, there was no significant difference in the variation of hemogram after splenectomy between the two groups (*P* > 0.05). Similarly, the variation of liver function indexes showed no statistical difference between the two groups. In terms of the incidence of postoperative complications including abdominal bleeding, pancreatic leakage, portal vein thrombosis treatment, incision infection, lung infection, and 36-month mortality, there were no significant differences between the two groups.

**Conclusion:**

After splenectomy, the hemogram as well as liver function in the HLD group improved a lot and showed a consistent tendency with that in the HBV group. Meanwhile, compared to the HBV group, there was no significant difference in the incidence of postoperative complications in the HLD group. All these results indicate that splenectomy in HLD patients combined with hypersplenism is completely feasible and effective.

## Introduction

Hepatolenticular degeneration (HLD) was first described by Wilson in 1912 and is also known as Wilson's disease (1). It is a recessive genetic disease caused by copper transporter gene ATP7B mutation and results in excessive copper deposition, especially in liver tissue (2). The incidence of this disease is between 1:30,000 and 1:100,000 (3). According to the affected organs, the clinical manifestations of hepatolenticular degeneration differ, including liver function injury, nervous system, and mental performance (4, 5). Since the liver is the main organ of copper metabolism, chronic liver diseases are often the most common manifestations in patients with HLD (5). Some patients also present with hemolytic anemia and impaired renal function (6, 7). When copper accumulates in the liver to a moderate level, it will not only cause liver cirrhosis but also portal hypertension, which eventually develops to splenomegaly and hypersplenism (8).

At present, the main strategy for medical treatment is to remove copper, and the commonly used drugs for removing copper are mainly chelating agents D-penicillamine and tetrathiomolybdate (9–12), whose joint toxic and side effects are to inhibit the decline of whole blood cells caused by the bone marrow. Because these kinds of patients with hypersplenism have complete hemopenia, often internal medicine to remove copper is difficult to maintain. HLD is a congenital genetic disease, which requires lifelong cuprous removal treatment to achieve the same life span, life, study, and work as ordinary people. When liver transplantation is limited due to the shortage of donors, splenectomy to restore blood cells is often a necessary choice (13). The purpose of this study is to further evaluate the postoperative risk of splenectomy for HLD based on the observation of whether the splenectomy can achieve benefits and to provide support for the widespread implementation of this technique.

Previous studies have shown that splenectomy for portal hypertension caused by viral hepatitis is safe and reliable. Until now, no studies have examined and evaluated patients with HLD hypersplenism who underwent splenectomy. Therefore, we attempted to evaluate the safety and effectiveness of splenectomy for HLD patients by observing the differences in postoperative blood routine, liver function, and postoperative complications between the two groups.

## Methods

### Clinical research design

**Figure 1:**
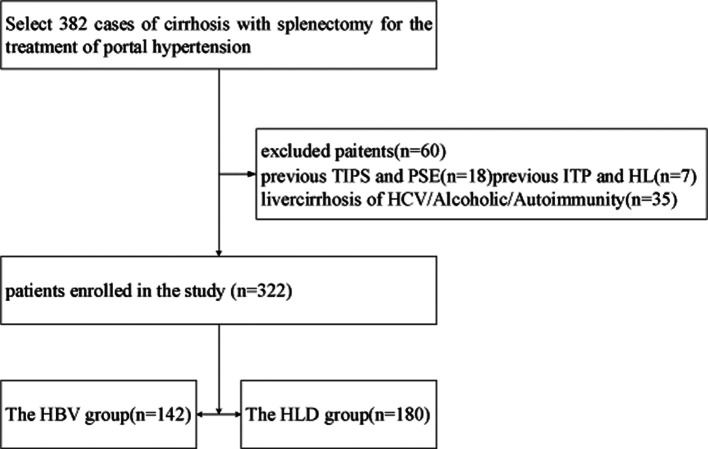
Diagram of the classification of patients who participated in this research.

The clinical data of patients diagnosed with HLD combined with hypersplenism and undergoing splenectomy in the Department of General Surgery of the First Affiliated Hospital of Anhui University of Traditional Chinese Medicine from January 2001 to December 2015 were retrospectively analyzed. There were 98 males and 82 females in the HLD group, aged 19–57 years. All patients were diagnosed with HLD and hypersplenism before surgery. Liver function grading: 115 cases were grade A and 65 cases were grade B. In the viral hepatitis B (HBV) group, 142 patients were diagnosed with HBV and hypersplenism at the age of 15–87 years. Liver function grading: 87 cases were grade A and 55 cases were grade B, as shown in Figure 1.

The study has been approved by the Ethics Committee of the First Affiliated Hospital of Anhui University of Traditional Chinese Medicine and complies with the Helsinki Declaration Batch (Batch No.: 2019AH-32). All participants signed informed consent before collecting data.

### Diagnostic criteria

(1) Diagnostic criteria for HLD. (a) Family genetic history: Parents are close relatives, compatriots with HLD patients, or those who die from unexplained liver disease. (b) Neuropsychiatric symptoms: slow progressive tremor, muscle stiffness, dyslexia, and liver symptoms. (c) Kayser–Fleischer ring on the cornea. (d) Ceruloplasmin level <1.6 μmol/24 h. (e) Liver copper concentration >250 μg/g (dry weight) (14).
(2) Diagnosis of hepatitis B. Based on clinical manifestations as well as serological and virological examinations (15).
(3) Child–Pugh grading criterion for liver function (16): serum bilirubin, ascites, serum albumin concentration, and prothrombin time were scored as 1, 2, and 3 according to different levels (mild level scored as 1, medium level as 2, and severe level as 3). Grade A was 5–6 scores, and the risk of surgery was low. Grade B was 7–9 scores, and the risk of surgery was medium. Grade C was 10–15 scores, and the risk of surgery was high.

### Inclusion and exclusion criteria

Inclusion criteria: (1) diagnosed with hepatolenticular degeneration; (2) diagnosed with hypersplenism by color ultrasound, blood test, and bone marrow puncture; (3) Child–Pugh grade was A or B; (4) all included patients are willing to undergo surgery.

Exclusion criteria: (1) patients combined with hematopoietic system diseases, cardiovascular and cerebrovascular diseases, and hepatic and renal serious primary diseases, which are intolerant to surgery; (2) patients with neurological symptoms.

### Therapeutic method

#### Preoperative preparation

Due to the suspension of normal anti-copper treatment during surgery, penicillamine and sodium dimercaptopropane sulfonate were applied to remove excess copper before surgery once the surgery date has been set. If the liver function still couldn't reach Child–Pugh grade A or B, transient use of glutathione, polyene phosphatidylcholine, and other liver-protecting drugs were applied. For those with abnormal coagulation function, 500 units of prothrombin complex were intravenously injected 30 min before surgery.

#### Anesthesia and surgical methods

All patients underwent open precise splenectomy. The specific surgical procedure is open the abdomen, separate the gastric colon and gastric spleen ligament, exposure and ligate the splenic artery, and autologous spleen blood reinfusion. Pull out the spleen with care, ligate the secondary and tertiary vessels at the upper and lower ends of the spleen one by one without blood, and then remove the spleen. While dividing the area around cardia, the high esophageal branches, collateral branches of paraesophageal veins, inferior phrenic branches, and peripheral vessels in the range of 6–8 cm in the lower esophagus were cut off. The wound was processed with hemostasis and suture serosa. Finally, a drainage tube was placed in the lower part of the spleen and the abdomen was closed layer by layer.

#### Postoperative treatment

(1) Conventional treatment: Postoperative anti-infection treatment, fluid replacement, nutritional support, hemostasis, and liver-protecting treatment.
(2) Treatment of postoperative complications. (a) Intraperitoneal hemorrhage treatment: Closely monitor the abdominal drainage and vital signs after surgery. Laparotomy is required under the condition that blood fluid in drainage is greater than 50 ml/h, hemoglobin level is less than 70 g/L with shock and other medical treatments are ineffective. (b) Pancreatic leakage treatment: Keep the drainage flow if complicated with fistula and grade B pancreatic leakage. Use somatostatin as appropriate if complicated with grade C pancreatic leakage. (c) Portal vein thrombosis treatment (PVST): Subcutaneous injection of low-molecular-weight heparin (LMWH) 4000 iu for 12 h, and urokinase 200 thousand bid. When thrombus ablation occurs and portal blood flow as well as platelet level are normal, keep the subcutaneous injection of LMWH or warfarin oral administration for 3–6 months.

### Observation index and detection method

(1) Blood routine detection: 5 ml of peripheral venous blood was extracted 1 day before the operation and 1, 3, 5, 7, and 14 days after the operation with an empty stomach. An automated blood cell analyzer (XN-9000, Sysmex) was applied to detect the level of white blood cells (WBC), red blood cells (RBC), and platelets (PLT) using the Coulter method.
(2) Liver function detection: 5 ml of peripheral venous blood was extracted 1 day before the operation and 1, 3, 5, 7, and 14 days after the operation with an empty stomach. An automatic biochemical analyzer (type 7600-010, Hitachi) was applied to detect alanine aminotransferase (ALT) and aspartate aminotransferase (AST) levels using the International Federation of Clinical Chemistry (IFCC) method, and total bilirubin (TB) levels with the Bromocresol Green (BCG) method.
(3) Postoperative complications and mortality:
  (a) Abdominal hemorrhage. Postoperative bleeding was defined as a decrease of hemoglobin over 20 g/L after surgery. Postoperative observation of the drainage fluid color and clinical manifestations, and was confirmed by color Doppler ultrasound and CT (17). The formula for estimating intraoperative blood loss is described as (18): the total amount of liquid in the suction reservoir + (the weight of blood gauze and the net weight of gauze and normal saline) − the amount of flushing liquid.
  (b) Pancreatic leakage. Pancreatic leakage was defined as the drainage fluid amylase being three times greater than serum amylase (19). Pancreatic leakage can be divided into three grades: grade A (biochemical leak)—only amylase is elevated without any clinical symptoms; grade B—clinical signs of infection and therapeutic measures should be changed; grade C—single or multiple organ dysfunction.
  (c) Portal vein system thrombosis (PVST). Digital color ultrasonic diagnostic apparatus (Prosound α6) was used to detect whether thrombosis was formed in the portal system (the main portal vein, intrahepatic branch, mesenteric vein, and splenic vein) (20).
  (d) Incision complications. Incision infection, bleeding, and dehiscence.
  (e) Pulmonary and urinary infection. Diagnostic criteria for pulmonary infection: body temperature >37.5°C, white blood cell count >10 × 10^10^/L, and neutrophils >90%. Pulmonary imaging or CT examinations were consistent with infection (21). Diagnostic criteria for urinary infection: postoperative bacterial culture was positive (22).
  (f) Mortality rate. Patients who died from surgery to discharge.

### Statistical methods

Data analysis was performed using SPSS21.0 statistical software. The measurement data was expressed as *x* ± *s*, and *t-*test was used when the data satisfy the normal distribution. Otherwise, the Wilcoxon rank sum test was used. Repeated measures of ANOVA or rank sum test (Mann–Whitney *U*) were used for the data of the two groups. The count data were expressed as the number of cases and percentages. The disordered classification data were analyzed by the *x*^2^ test. The difference was considered to be statistically significant when *P* < 0.05. The end point time was defined as the period from the date of surgery to the date of death; otherwise, it was defined as censored data, calculated by Kaplan–Meier method using survminer and survival in R language, and performed log-rank test and plotted using ggplot.

## Results

### Demographic characteristics

Data of 142 inpatients with hepatitis B and hypersplenism and 180 in patients with HLD and hypersplenism were analyzed. The mean age, sex, course of disease, and liver function grade of these patients are shown in Table 1.

**Table 1:** Comparison of general data of the two groups of patients.

Project	HLD (*n* = 180)	HBV (*n* = 142)	Statistics	*P* value
Gender [male/female, patients (%)]	98 (54)/82 (46)	81 (57)/61 (43)	*x*^2^ = 2.14	0.14
Age (year)	47.47 ± 11.25	46.58 ± 13.41	*t* = 0.65	0.51
Child–Pugh [A/B, patients (%)]	115 (63)/65 (37)	87 (62)/55 (38)	*x*^2^ = 0.23	0.62
BMI (kg/m^2^)	24.15 ± 5.94	24.59 ± 5.89	*t* = −0.60	0.54
Diabetes [Y/N, patients (%)]	40 (22.2)/140 (78.2)	34 (23.9)/108 (76.1)	*x*^2^ = 0.13	0.71
Hypertension [Y/N, patients (%)]	33 (18.3)/147 (81.7)	22 (15.5)/120 (84.5)	*x*^2^ = 0.45	0.50
Ascites [Y/N, patients (%)]	68 (37.8)/112 (62.2)	45 (31.7)/97 (68.3)	*x*^2^ = 1.29	0.25
Portal vein diameter (mm)	15.61 ± 1.59	15.23 ± 2.27	*t* = 1.75	0.08
Splenic vein diameter (mm)	6.22 ± 1.76	6.16 ± 1.97	*t* = 0.29	0.76
Operation time (min)	210.18 ± 16.51	205.90 ± 27.50	*t* = 1.73	0.08
Intraoperative blood loss (ml)	209.40 ± 17.46	206.57 ± 25.93	*t* = 1.16	0.24

HLD, hepatolenticular degeneration; HBV, viral hepatitis B; BMI, body mass index.

### Blood routine before and after splenectomy in two groups

**Figure 2:**
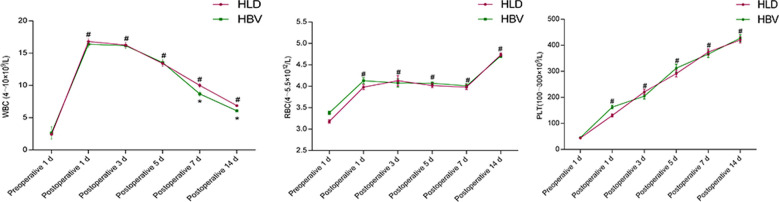
Blood routine before and after splenectomy in two groups. # refers to *P* < 0.05 compared with preoperative, * refers to the difference between the two groups at each time point *P* < 0.05, and ** refers to the difference between the two groups at each time point *P* < 0.01.

By comparison, the white blood cell count, red blood cell count, and PLT count of the two groups were higher after the operation than before the operation (*P* < 0.05), and there was no difference in the WBC count between the two groups on the first, third and fifth day after operation (*P* > 0.05). On the 7th and 14th day after operation, the WBC count of HLD patients was higher than that of the HBV group (*P* < 0.05). The RBC count of the HLD group was significantly different from that of the HBV group 1 day after the operation (*P* < 0.05), but there was no difference at 3, 5, 7, and 14 days after the operation (*P* > 0.05). There was no difference in PLT count between the two groups on postoperative days 1, 3, 5, 7, and 14 (*P* > 0.05) (Figure 2).

### Comparison of liver function before and after splenectomy in two groups

The ALT counts of the two groups were increased on the 1st and 3rd day after operation compared with those before operation (*P* < 0.05), and decreased on the 14th day after operation compared with those before operation (*P* < 0.05). There was no significant difference between the two groups at each time point (*P* > 0.05).

The AST count of the two groups was increased on the 3rd day after operation compared with that before operation (*P* < 0.05), and decreased on the 7th and 14th day after operation compared with that before operation (*P* < 0.05), and there was no significant difference between the two groups at each time point (*P* > 0.05).

**Figure 3:**
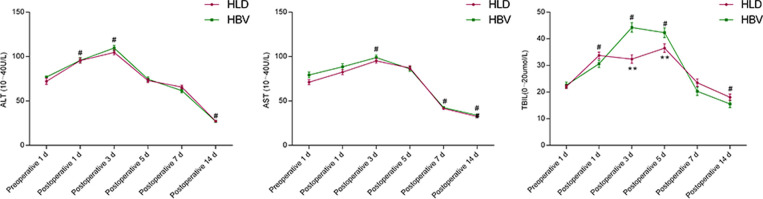
Comparison of liver function before and after splenectomy in two groups. # refers to *P* < 0.05 compared with preoperative, * refers to the difference between the two groups at each time point *P* < 0.05, and ** refers to the difference between the two groups at each time point *P* < 0.01.

The Total Bilirubin (TBIL) count of the two groups was increased on the 1st, 3rd, and 5th day after operation compared with that before operation (*P* < 0.05), and decreased on the 14th day after operation compared with that before operation (*P* < 0.05). There was a difference between the two groups on the 3rd and 5th day after operation (*P* < 0.05). There was no significant difference between the two groups on postoperative day 1 and day 14 (*P* > 0.05) (Figure 3).

### Postoperative complications after splenectomy in two groups

As shown in Table 2, there was no difference in complications between the two groups (*P* > 0.05): one patient in the HLD group died due to ascites and liver function failure caused by portal vein thrombosis, and one patient in the HBV group died due to abdominal hemorrhage after surgery.

**Table 2:** Comparison of complications after splenectomy in the two groups.

Complications	HLD (*n* = 180)	HBV (*n* = 142)	P^N^
Abdominal bleeding	2	2	0.81
Pancreatic leakage	7	6	0.87
PVST	100	78	0.91
Incision infection	2	2	0.81
Pulmonary infection	3	3	0.76
Urinary tract infection	0	0	—

P^N^ refers to the postoperative time of main and in vivo effects in the group × the fixed time level of the group, and the differences in each level of individual effects in the group.

HLD, hepatolenticular degeneration; HBV, viral hepatitis B; BMI, body mass index; PVST, portal vein thrombosis treatment.

### The two groups were followed up after splenectomy

**Figure 4:**
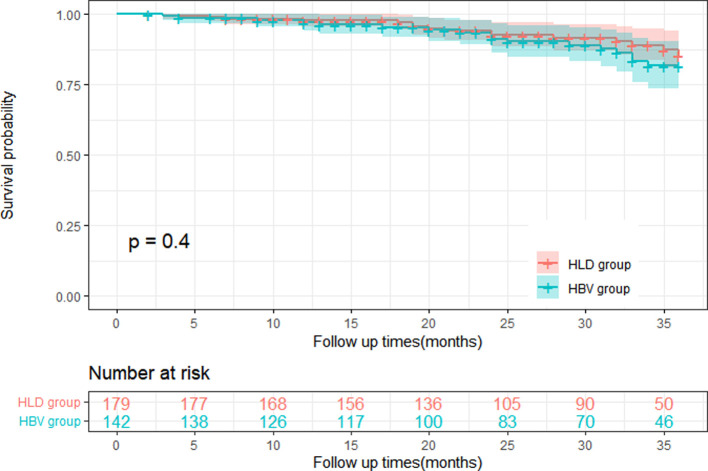
Comparison of mortality between two groups after 36 months of follow-up.

After 36 months of follow-up, the mortality of HLD and HBV patients did not exceed the median survival time, and the postoperative follow-up cutoff survival rate of the two groups was 85.2% and 81.6%, the difference was not statistically significant (log-rank = 0.702; *P* = 0.400), as shown in Figure 4.

## Discussion

The incidence of HLD in China is around 6/100,000, and our department is the largest HLD treatment center in China (23–28). In the beginning, we performed splenectomy in the pursuit to relieve hypersplenism, normalize blood cells level, and meet the requirement of lifelong anti-copper treatment. We have previously reported our experience and results in this regard (23–28). The expanded sample size of HLD patients involved in this work further confirmed that splenectomy can achieve the desired anti-copper effect and improve the blood cells level.

In the present observation, we have the following new findings: (1) RBC level increased a little after surgery, but the change was not statistically significant; (2) the level of WBC increased gradually from 1 to 7 days after splenectomy and decreased to the normal range from 7 to 14 days after splenectomy; (3) the level of PLT gradually increased from 1 to 14 days after surgery and reached a peak. Attention should be paid because there is a possibility of PVST formation if the PLT level is higher than 500 × 10^9^/L. Generally, our clinical experience is continuously monitoring the level of PLT and D-dimer, meanwhile detecting PVST with digital color ultrasound. If the PLT level is higher than 500 × 10^9^/L, then prophylactic anticoagulation treatment is needed. The detailed procedure was as follows: subcutaneous injection of low-molecular-weight heparin 4000 iu for Q12 h, and they maintain the treatment for 2–3 weeks after the PLT level returned to normal.

Except for the improvement of blood cells level, we have also noted that liver function was enhanced to varying degrees after splenectomy in HLD patients. Several different arguments are trying to clarify the underlying mechanism. Some studies focused on the secretion of IL-1, IL-6, TNF, TGF-β, and other cytokines due to splenomegaly, which leads to the formation of cirrhosis. Splenectomy can remove these cytokines and reduce inflammatory damage, thus promoting liver blood supply, and contributing to liver cell regeneration as well as resultant liver function improvement (29, 30). However, we are more convinced that the enhanced liver function is related to splenic artery steal syndrome, which refers to splenomegaly, splenic artery enlargement and thickening, blood flow acceleration, and other pathophysiological changes in patients with portal hypertension. Since both the splenic artery and the hepatic artery originate from the celiac trunk artery, the enlarged splenic artery and increased blood flow in the spleen will compete with the hepatic artery for the blood flow from the celiac trunk, resulting in the narrowing of the hepatic artery and the decrease of hepatic blood flow. Those changes ultimately result in an insufficient blood supply of liver tissue (normally more severe based on cirrhosis), liver cell damage, and hepatic dysfunction. After splenectomy, both hepatic blood supply and liver function will be promoted because of increased blood in the hepatic artery (31, 32).

The results of our study showed that the level of ALT and AST increased gradually 1–3 days after splenectomy, and decreased to normal 7–14 days after splenectomy. The level of TB increased gradually 1–5 days after splenectomy and decreased to normal 7–14 days after splenectomy. Up to now, literature studies about splenectomy and liver function change are mainly focused on cirrhosis patients due to hepatitis or other etiology (33, 34), and few studies have been reported on liver function change after splenectomy in HLD patients. The present work can enrich relevant data in this field.

It has been reported that the mortality rate after splenectomy is 1.1%–1.63% (35, 36), and the complication rate is 12.9%. In our study, the mortality rate after splenectomy in the HBV group and the HLD group was 0.7% and 0.6% respectively, and there was no statistical difference in the mortality rate between the two groups. The incidence of postoperative complications in our results was higher than reported in literature studies because we focused on the complication of portal vein thrombosis. Notably, splenectomy in HLD patients does not result in increased mortality and is safe and feasible with relatively low surgical risk. In terms of postoperative complications and corresponding treatment, we would like to note: (1) hemorrhagic complications – the incidence of abdominal hemorrhage after splenectomy was 1.19% (37) as reported in the literature. This incidence was 1.4% in the HBV group and 1.11% in the HLD group, indicating that splenectomy in HLD patients will not increase the risk of bleeding. In this study, the incidence of abdominal hemorrhage in the HLD group was even lower than that reported in the literature and the HBV group, which may be related to (a) preoperative liver protection and prothrombin complex were used to improve the coagulation function, (b) intraoperative autologous splenic blood transfusion, making a large number of coagulation factors entering the body; precise splenectomy that performed during the operation also remarkably reduced the risk of bleeding, (c) postoperative application of liver protection and prothrombin complex to enhance the coagulation function. (2) Pancreatic leakage. Previously, we reported that the incidence of pancreatic leakage after splenectomy was 4.2% (28), as compared to 3.88% in the present study. The incidence of pancreatic leakage in HBV group and HLD group was 4.2% and 4.1%, respectively. The risk of pancreatic leakage was not increased after splenectomy in HLD patients. The treatment of pancreatic leakage differs according to varying situations. If complicated with fistula and grade B pancreatic leakage, then keeping the drainage flow is enough, and no need to use drugs for inhibiting pancreatic secretion. For patients with grade C pancreatic leakage and large drainage volume, somatostatin should be used appropriately. (3) (a) Complication of PVST. The incidence of PVST has been reported to be 24.6% (38) after splenectomy. In this study, the incidence of PVST in HBV and HLD groups was 54.92% and 55.55%, respectively. (b) Hazard of PVST. According to our observation, the hazard of PVST varies with the site of occurrence. The formation of complete PVST in the main portal vein or intrahepatic branch can not only show abnormal liver function indicators but also show clinical manifestations such as jaundice, ascites, hypoproteinemia, difficulty in wound healing or even incision dehiscence, which should be paid great attention to. PVST is formed in the main part after intrahepatic branches, transaminase, and jaundice are often transient. The partial thrombosis in the mesenteric vein, with only abdominal distension, abdominal pain, decreased digestive function, and other gastrointestinal symptoms, is easily ignored or misdiagnosed as the gastrointestinal function has not been fully recovered after surgery. If complete obstruction occurs, intestinal congestion, intestinal obstruction, intestinal bleeding, and, in severe cases, intestinal necrosis and perforation. Splenic vein thrombosis is a common fever; we used to think of spleen fever as mostly caused by severe splenic vein thrombosis. Since the splenic vein formed after splenectomy is blind, it is usually not harmful to the body. (c) Prevention and treatment of PVST. It is found that there are high-risk factors for PVST formation, such as splenomegaly, portal vein diameter widened by preoperative examination, severe surgical trauma or traditional splenectomy, postoperative platelet elevation, and slow portal vein blood flow, and usually, preventive measures should be taken (39, 40). Based on the above data, the risk of postoperative PVST was not increased in the HLD group. Once PVST occurs, subcutaneous injection of low-molecular-weight heparin (LMWH) should be applied with a dose of 4000 iu for 12 h, and urokinase 200 thousand bid. When thrombus ablation happens and portal blood flow as well as platelet level are normal, keep the subcutaneous injection of LMWH or warfarin oral administration for 3–6 months. (4) Incision complications: It has been reported that the incidence of incision infection caused by splenectomy is 4% (41). In our study, the incidence of incision infection in the HBV group and the HLD group was 1.4% and 1.1%, respectively. Those results suggested that the risk of postoperative incision complications was not increased in HLD patients after splenectomy. To prevent HLD incision dehiscence, our experience is extending stitches removal to 12–14 days postoperatively. (5) Complications of systemic infection (lung and urinary tract). Literature studies reported that the incidence of pulmonary infection after splenectomy was 3.8% (37), and the incidence of urinary tract infection was 0.21% (42). In our study, the incidence of splenectomy for the HBV group was 2.11%, and the incidence of splenectomy in HLD was 1.66%. Based on the above data, splenectomy in HLD does not increase the risk of systemic infection complications. If systemic infection indeed happens, the assistance of a physician is usually needed. At 36 months of follow-up after splenectomy, there was no increase in postoperative mortality in HLD patients with hypersplenism. Although our finding has indicated that splenectomy for HLD complicated with hypersplenism is feasible and beneficial, the underlying molecular mechanism of liver function improvement, therefore, needs to be further studied through cell and animal experiments. Owing to our research being a single-center retrospective analysis, it is necessary to expand the sample size and conduct multicenter verification in the future.

## Conclusion

Splenectomy in HLD patients combined with hypersplenism achieved the expected effects of enhancing blood cells and improving liver function. There was no increased risk of postoperative complications compared with splenectomy for HBV patients in the same period. Therefore, we conclude that splenectomy for HLD with hypersplenism is safe and feasible.

## Data Availability

The original contributions presented in the study are included in the article/Supplementary Material, further inquiries can be directed to the corresponding author.
